# An optical-magnetic Material as a toxic gas filter and sensing device

**DOI:** 10.1039/d0ra00537a

**Published:** 2020-06-17

**Authors:** Thuanny Almeida Moraes, Maria Julia Farrôco, Ketly Pontes, Magda Fontes Bittencourt, Bluma Guenter Soares, Fernando Gomes Souza

**Affiliations:** Macromolecules Institute: Professor Eloisa Mano, Technology Center-University City av. Horácio Macedo, 2030, block J. Federal University of Rio de Janeiro RJ Brazil fernando_gomes@ima.ufrj.br; Department of Metallurgical and Materials Engineering, COPPE, Technology Center-University City av. Horácio Macedo, 2030, bloco F. Federal University of Rio de Janeiro RJ Brazil; Brazilian Center for Physical Research, CBPF Rua Dr. Xavier Sigaud, 150 - Urca Rio de Janeiro – RJ Brazil; Nanotechnology Engineering Program, COPPE, Technology Center-University City av. Horácio Macedo, 2030, bloco F. Federal University of Rio de Janeiro RJ Brazil

## Abstract

The objective of this work is the development of a toxic gas detector/filter based on the production of porous polyaniline composites filled with magnetic nanoparticles. The composite produced was subjected to hydrogen sulfide gas as a preliminary test of its detection and sorption capacity, which were proven by gravimetric analysis. Analysis by light scattering and TEM indicated that magnetic nanoparticles with a size of approximately 5 nm were obtained through the proposed methodology. FTIR spectroscopy, UV-vis spectroscopy, TGA, and DSC were performed to prove the successful synthesis of the composite. To identify the specific properties of each constituent of the composite, the conductivity and magnetic force of the material were determined. The SEM results showed that the morphology was useful for the sorption process with the formation of pores in the polymer matrix, allowing the percolation of the gas for splicing by the nanoparticles. TGA, electrical conductivity, magnetic force, UV-vis spectroscopy, and EDS analyses were also performed after the detection/sorption tests to demonstrate the functioning of the material.

## Abstract 2

The objective of this work is the development of a toxic gas detector/filter based on the production of porous polyaniline composites filled with magnetic nanoparticles.

## Graphical abstract

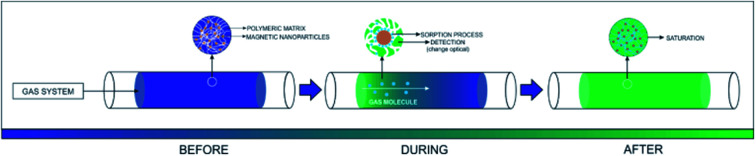

## Introduction

Since its discovery 180 years ago,^1^ polyaniline (PAni) has been reported in the literature as an intrinsically conductive polymer with great technological and scientific applicability due to its peculiar properties such as excellent thermal and environmental stability, ready availability and excellent electrical and optical properties.^2,3^ As a sensor, PAni has received significant attention^4^ since its different oxidation states allow changes in its optical properties when subjected to different acidic or alkaline analytes^5^ as well as its electrical properties.^6^

Given the excellent properties and advantages of PAni, the combination of this polymer with different materials has been the target of many studies to potentiate such properties as well as to form more efficient materials.^7,8^ The combination with inorganic materials, in particular oxides, has created a class of desirable polymer/metal materials for technological applications.^9,10^ The composites or hybrids of PAni and graphene or iron oxides, for example, have been the basis for the development of polymers with better conductive and magnetic properties.^8,11^

Magnetic particles, especially iron oxide (Fe_3_O_4_) nanoparticles, have been widely reported in the literature^12^ in applications in the area of cancer treatment with magnetic hyperthermia, water treatment,^13,14^ catalysts,^15^ and heavy metal adsorbers^16–18^ besides their use in the adsorbers of toxic gases.^19^ Their viability is due to their facile synthesis, good yields, and high sorption capacity as a result of the electrostatic interactions that contribute to excellent adhesion as well as the possibility of binding with macromolecules and functional groups on the surfaces of the magnetic particles.^20^

In this context, this work has the premise of obtaining PAni composites filled with iron oxide nanoparticles for the development of a toxic gas filter/sensor. The nanoparticles were previously obtained from a colloidal dispersion of polyvinyl alcohol (PVA) in order to obtain a suitable nanoscale size as well as promote a morphology conducive to the sorption of the composite. For the application of the proposed material, adsorption and detection tests were performed in the presence of hydrogen sulfide.

## Experimental section

### Materials

All chemicals were of analytical grade. Polyvinyl alcohol with a degree of hydrolysis of 89% (molecular weight, *M*_w_, of about 115.000 g mol^−1^, Sigma-Aldrich), ferrous chloride (FeCl_2_·4H_2_O (Sigma-Aldrich)), ferric chloride (FeCl_3_·6H_2_O) (Sigma-Aldrich), sodium hydroxide (NaOH) (Sigma-Aldrich), deionized water, aniline (Sigma-Aldrich), dodecylbenzene sulfonic acid (DBSA), and ammonium persulfate ((NH_4_)_2_S_2_O_8_) (Sigma-Aldrich) were used without further purification.

### Synthesis of magnetic nanoparticles

Magnetic nanoparticles were prepared following the methodology proposed by Souza Jr and co-workers.^6^ For the procedure, 1 L of an aqueous solution using different concentrations (1%, 2% and 5%) of PVA deionized water was initially prepared with mechanical stirring at 50 °C for 1 hour.

PVA with different concentrations was used to identify the PVA/NPIO dispersion that formed the smallest size of nanoparticles.

FeCl_2_ and FeCl_3_, in a molar ratio 1 : 2, were then added to the 5% PVA solution. The solution was kept under stirring for 1 hour at room temperature to dissolve the added salts. Fe^2+^ and Fe^3+^ ions were then co-precipitated with 30 mL of 1 molar NaOH solution, added slowly, to obtain the magnetic nanoparticles. The magnetic nanoparticles were kept under mechanical agitation for 1 hour at room temperature. The obtained colloidal dispersion was centrifuged, with two cycles of 30 minutes each, at 4000 rpm at room temperature. For comparative purposes, magnetic particles were synthesized by co-precipitating the Fe^2+^ and Fe^3+^ in an aqueous medium, in place of the PVA dispersion.

### Synthesis of PAni and magnetic nanoparticle composites

The synthesis of PAni was performed following the procedure described elsewhere by our group.^20^ The synthesis of the composites was carried out by the *in situ* polymerization of PAni in the presence of the colloidal dispersion of nanoparticles. In the process, 100 mL of a DBSA solution, 1 mol L^−1^, was first prepared, which was maintained under mechanical stirring and room temperature for 1 h. Then, the colloidal nanoparticle dispersion (NPOI), with a concentration of 20%, previously measured by TGA, in which an amount of 0.53 mg of magnetic nanoparticles was contained in 20 μL of the dispersion, was added and kept in the shaking system for 1 hour. This was followed by the *in situ* polymerization of PAni, where aniline, 1 mol L^−1^, was added to the system and after 1 hour, 20 mL of an acid solution of the oxidizing agent, ammonium persulfate, 1 mol L^−1^, was slowly dripped into the reaction, which was maintained under mechanical stirring and at room temperature for 1 hour. After the reaction, the material was centrifuged with successive washings with alcohol, until the supernatant was colorless, to remove excess PVA to obtain the proposed morphology. The composite was obtained as a powder, PAni/NPOI 20%, and was dried at room temperature for 24 hours.

### Exposure of the material to toxic gases

First, 0.5 g of each sample was inserted into the center of 7.00 × 0.75 (*L* × *D*) plastic tubes of approximately 0.66 g, which were discounted for gravimetric analysis.

The gas production and exposure system consisted of a Kipp pipette coupled to a 250 mL Erlenmeyer flask containing FeS and HCl in the 2 : 1 molar ratio and the system was heated to approximately 50 °C. The plastic tubes containing the samples were connected to the Kipp pipette at one end and the other to a gas collection and identification system through a glass pipette. The collection and identification system consisted of a 250 mL round bottom flask with three openings. In the first opening, the pH meter electrode was connected to determine the variation of the gas concentration at the inlet and outlet; in the second opening was the plastic tube containing the sample, and the third end was sealed. The tube was weighed every 15 minutes, over 1 h, to evaluate the mass variation during exposure.

## Characterization

### Dynamic light scattering (DLS)

The dispersions of magnetic nanoparticles in 1%, 2% and 5% PVA were characterized using a particle size analyzer, the Zetasizer Nano ZS Malvern, in order to obtain the size range of the particles dispersed in the colloidal dispersion of PVA and their size distribution. The equipment had a detector at 173° from the incident light beam, known as backscatter detection, patented technology known as NIBS using a refraction index of 1.700, and a magnetite correspondent under room temperature conditions, with 20 scans in triplicate. For this analysis, 1 drop of the sample was diluted in 1 mL PVA solution, with the corresponding concentration, and inserted in a quartz bucket. The data were treated with a Qtiplot 0.9.8.6 program.

### Transmission electron microscopy – TEM

The analyses were carried out at the Center for Structural Biology and Bioimaging – CENABIO, using an FEI Tecnai Spirit Electronic Transmitting Microscope with 120 kV acceleration voltage and tungsten filament with a 2k Veleta Olympus digital camera. For analysis, 20 μL of the magnetic nanoparticle dispersion (NPIO/PVA5%) was diluted in 10 mL of 5% PVA solution and dripped onto a formvar-coated copper grid with 200 mesh spacing.

### Thermogravimetric analysis and differential scanning calorimetry – TGA and DSC

The tests were performed using a TA Instruments TGA Q500-V6.7 Build 203 under an inert atmosphere of nitrogen (purge gas – 19.8 mL min^−1^), at temperatures between 30 °C and 700 °C and a heating rate of 20 °C min^−1^. In a typical test, 20 μL of the sample, measured with the aid of an automatic micro-pipette, was used for the analysis. Tests were performed five times and the 95% confidence limits were calculated. The data were treated with a Qtiplot 0.9.8.6 program.

### Scanning electron microscopy and energy dispersive X-ray – SEM and EDX

This analysis was carried out in partnership with the Mineral Technology Center (CETEM). For the tests, the samples in powder form were carbon-plated and placed in a JEOL model JSM-5610LV under a 20 kV acceleration voltage with secondary electron detectors at magnifications of 100, 2000 and 5000 times.

### Atomic force microscopy

The morphology (topography, amplitude, phase) of the surface of the samples was analyzed by AFM (HITACHI 5100N). On a mica surface, the sample was deposited in the solid state. The evaluation was performed intermittently at room temperature. Silicon tips of Atomic Force Microscopy HA_NC coupled to the cantilever of length 117 μm, with a resonance frequency of about 110 kHz and a constant nominal force of 2 N m^−1^ were used. The images obtained were processed using the AFM5000 II Software.

### Electrical tests

For the determination of electrical conductivity, the two-electrode method was used according to the analyses already carried out in our research group at the Laboratory of Biopolymers and Sensors (LABIOS). For this, the samples were placed between the two electrodes. An electric voltage of 5 V was generated between these electrodes, which allowed the determination of the current that would cross the sample and the calculation of the conductivity of the materials. Conductivity tests were performed using a Keithley 6517B electrometer. The thicknesses of the samples were determined with the aid of a Digimess digital caliper. The electrical conductivity data, taken over two minutes of the experiment, were stored and used to calculate the resistivity.

### Magnetic force analysis

Magnetic force tests were performed following a procedure reported elsewhere by our group.^20^ The experimental setup was composed of an analytical scale (Shimadzu AY-220), a voltage source (ICEL PS-4100), a digital multimeter ICEL MD-6450, a Gaussmeter GlobalMag TLMP-Hall-02, a home-made sample holder and a home-made electromagnet. The electromagnet was made with a solid 12 cm long iron core with a 1.6 cm diameter circular section, and 4200 turns of 18AWG copper wire. The data were treated with the Qtiplot 0.9.8.6 program.

### Magnetization analysis

The magnetization tests were performed using a magnetometer from Quantum Design PPMS® VersaLab™. Tests were performed in partnership with the Magnetometric Laboratory of the Brazilian Center for Physical Research. The analyses were carried out at room temperature, with an oscillating magnetic field from −30 kOe to +30 kOe. The samples were inserted in a plastic sample-holder with a mass of around 7.5 mg. The data were treated with the Qtiplot 0.9.8.6 program.

### The BET (Brunauer–Emmett–Teller) method – multimolecular adsorption analysis – ASAP

To identify the sorption and desorption behavior of materials, we used the Adsorption Multimolecular Micromeritics ASAP 2010 analysis equipment with N_2_ gas 5.0 Analytical (White Martins) and an H_2_ tablet saturation tube (White Martins). The magnetic particles, PAni and the composite magnetic nanoparticles (20%), and PAni were used at treatment temperatures of 150 °C, 350 °C, and 120 °C, respectively. The data were treated with the Qtiplot 0.9.8.6 program.

### Gravimetric analyses

For gravimetric analyses, the samples were weighed on an analytical digital scale. They were then placed inside the system with gas production. Every 15 minutes, the samples were removed from the system, sealed and weighed to evaluate their mass variation until 60 minutes of exposure to the gas had been completed. Simultaneously, the pH of the system was measured to verify the amount of produced gas, in ppm. The data were treated with the Qtiplot 0.9.8.6 program.

## Results and discussion

### Colloidal dispersion of magnetic nanoparticles

#### Dynamic light scattering (DLS)

**Fig. 1:**
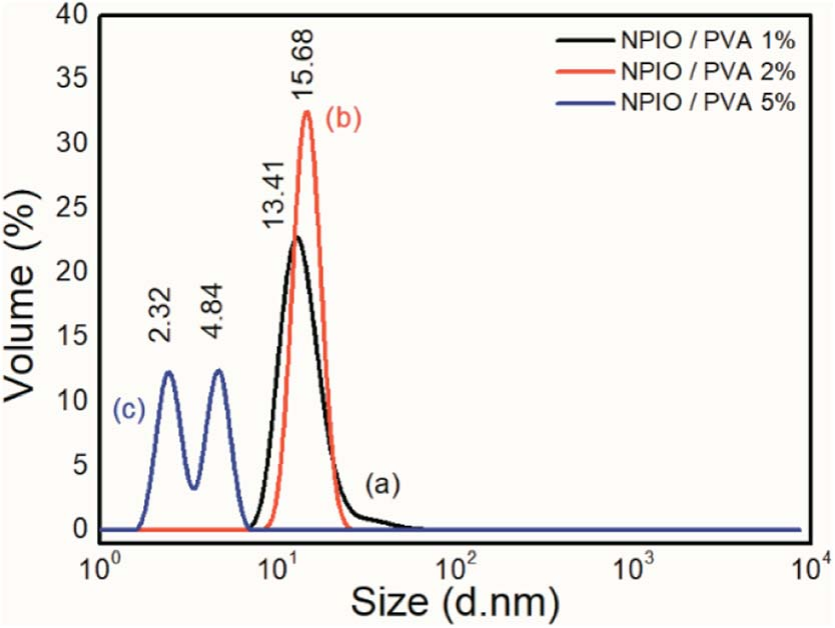
Size distribution analysis of the colloidal dispersions of magnetic nanoparticles in PVA at 1% (a), 2% (b) and 5% (c).

Fig. 1 shows the dynamic light scattering analysis to determine the size and distribution of the magnetic nanoparticles prepared using 1%, 2%, and 5% PVA concentrations.

Particle size distribution analyses were performed to identify the concentration of PVA at which the smaller-sized magnetic nanoparticles were obtained. It was observed that for the colloidal dispersion of magnetic nanoparticles containing 5% PVA, the smaller nanoparticle sizes between 2.32 nm and 4.84 nm were obtained with volumes of 13.50% and 14.03%, respectively.

The decrease in the diameter of the Fe_3_O_4_ nanoparticles as a function of the increase in the amount of PVA was attributed to the increase in the dispersion stability,^17^ avoiding the agglomeration of nanoparticles.^21,22^ This effect had already been proven in the synthesis of nanoparticles of Fe_3_O_4_, with average diameters between 4 and 7 nm and with super magnetic properties.^23^

### Thermogravimetric analysis (TGA) and differential scanning calorimetry (DSC)

**Fig. 2:**
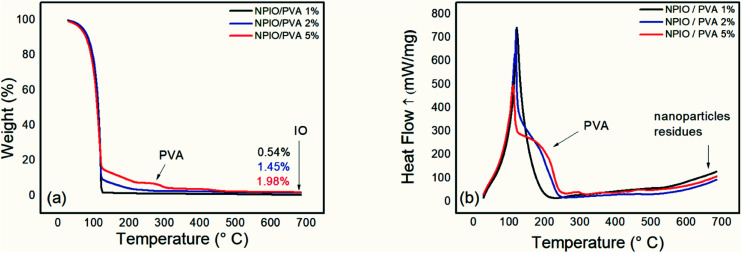
Thermogravimetric analysis (a) and differential scanning calorimetry (b) for the colloidal dispersion of magnetic nanoparticles in PVA at 1%, 2% and 5%.

Fig. 2(a) shows the thermogram of magnetic nanoparticle dispersions with 1%, 2%, and 5% PVA.

Thermogravimetric analysis was conducted to identify the residual mass present in the colloidal dispersions of magnetic nanoparticles, as well as natural degradations of the samples. In all the dispersions, the first thermal degradation event was observed up to 126 °C and was attributed to water loss.

A second thermal degradation event was observed at around 266–307 °C, which can be attributed to PVA decomposition.^14,16^ An increase in mass loss in this temperature range was observed as the PVA concentration in the dispersion of the magnetic nanoparticles was increased, proving the increase in the polymeric material in the dispersion. Such a system containing 5% PVA in the dispersion prevents the decanting of magnetic nanoparticles, whose supernatant contains the most significant number of nanoparticles. For the dispersion containing 5% PVA, a mass loss of approximately 3% was observed as being the most evident.

### Transmission electron microscopy – TEM

**Fig. 3:**
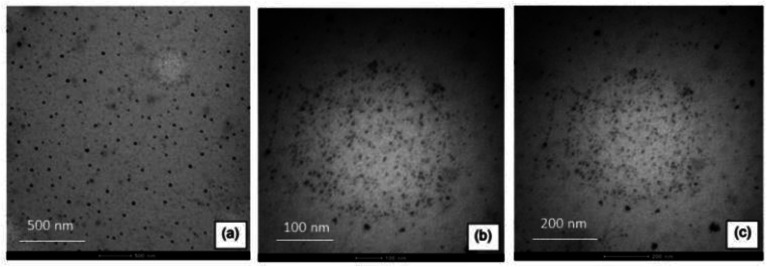
TEM micrographs of the colloidal dispersion of 5% PVA nanoparticles at (a) 500 nm, (b) 200 nm and (c) 100 nm resolution.

Fig. 3 shows micrographs of the colloidal dispersion of magnetic nanoparticles in PVA at 5%, with resolutions of 500, 200, and 100 nm.

The diameters of the Fe_3_O_4_ nanoparticles were confirmed by TEM. A good dispersion of nanoparticles with spherical morphologies was observed, thus confirming that the synthesis in the presence of 5% PVA prevented the agglomeration of the magnetic nanoparticles.^20^ Average diameters were found to be around (43.95 ± 1.24), (17.80 ± 0.79) and (6.95 ± 1.06), which are considered as nanoparticles according to the literature,^18,25^ and favour the sorption process.^15,21,26^ Droplets that had nanoparticles of different sizes were also observed, but these were below 6.95 nm, as shown in Fig. 3(c).

The third event, at around 300–450 °C, was attributed to the elimination of all PVA on the surface of the nanoparticles of Fe_3_O_4_. The thermal degradation of the PVA ultimately proved the adsorption of the polymer by the surface of the inorganic material through the hydrogen bonds of the polar functional groups in the PVA and the iron oxide.^23^ Thus, the dispersion of magnetic nanoparticles in PVA at 5% presented the best conditions for the proposed application. Therefore, this concentration was adopted for the synthesis of the composites since the concentration of PVA used prevented the agglomeration of the nanoparticles of Fe_3_O_4_ caused by the particle–particle interaction and its excellent surface energy.^24^

### PAni composites filled with magnetic nanoparticles

#### Fourier-transform infrared spectroscopy – FTIR

**Fig. 4:**
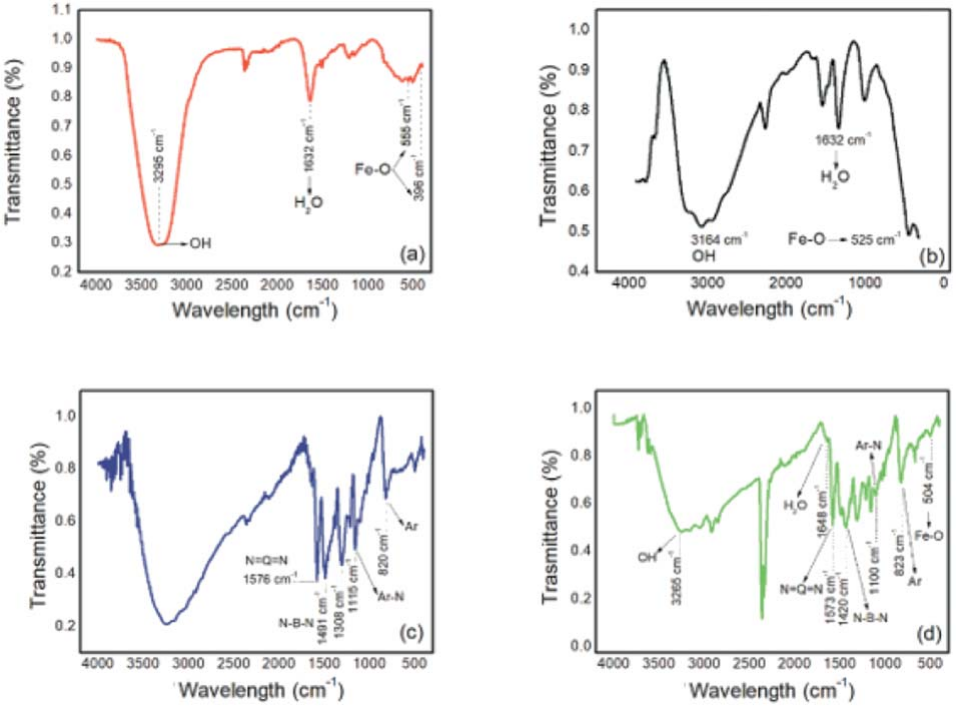
Infrared spectra of (a) NPIO, (b) IO, (c) PAni and (d) PAni/NPIO 20%.

The chemical structure of the materials, as well as the confirmation of the synthesis of the composite of PAni and magnetic nanoparticles, were investigated by Fourier transform infrared spectroscopy (FT-IR). Table 1 shows the absorptions of the insulating materials and the composite, with their respective wavelengths. Fig. 4 shows the absorption bands of each material.

**Table 1:** Characteristic absorption bands of the prepared materials^a^

Sample	Wavelength (cm^−1^)						
	Fe–O	Ar	Ar–N	N–B–N	N=Q=N	H_2_O	OH
IO	396/555	—	—	—	—	1632	3295
NPIO	525	—	—	—	—	1632	3164
PAni	—	820	1115	1308/1491	1576	—	—
PAni/NPIO	403/504	823	1110	1420	1573	1648	3265

a Ar is aromatic.

Absorbances at wavelengths of 555 cm^−1^ and 396 cm^−1^ were attributed to Fe–O,^27^ and absorptions at 3295 cm^−1^ and 1632 cm^−1^ were attributed to the vibrations of the H_2_O and OH groups, respectively, corresponding to the magnetic nanoparticles.^28^ These absorptions were also observed on the magnetic particles whose analyses were performed for comparative purposes. For pure PAni, absorptions were observed at around 1600 cm^−1^ and 1500 cm^−1^ and were attributed to the quinoid groups (N=Q=N) and benzoids (N–B–N), respectively, present in PAni.^29^ Other absorptions located around 1110 cm^−1^ and 820 cm^−1^ can be found in the structure of PAni and can be attributed to the vibrations of the aromatic rings and vibrations of the bonds between the nitrogen and the aromatic rings.^30^ For composite Fig. 4(c), absorptions were observed corresponding to both materials, thereby confirming the obtained PAni/NPIO 20% composite.

### Morphological studies

The morphological analyses by SEM and AFM were performed to identify the formation of pores in the polymer matrix in order to facilitate the transport of gas to the magnetic nanoparticles, allowing their sorption.

### Scanning electron microscopy – SEM

**Fig. 5:**
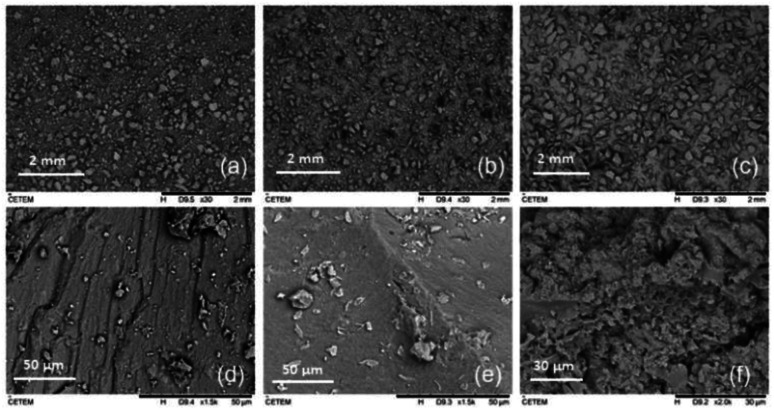
SEM micrographs of (a) magnetic particles, (b) PAni and (c) PAni/NPIO 20% composites with a magnification of 30 times. (d) Magnetic particles, (e) PAni and (f) PAni/NPIO 20% with a magnification of 1500 and 2000 times.

Fig. 5 shows micrographs of the samples of iron oxide particles, the pure PAni, and the composite.

The SEM analysis could demonstrate the presence of a porous structure for the composite of PAni and the magnetic nanoparticles as compared with micrographs of the isolated materials, which had a smooth surface morphology.

The morphologies of the magnetic particles and PAni have already been proven in different studies,^31^ whose images showed materials with smooth surfaces. For PAni,^32^ in addition to its smooth structure, grooves were observed, which can be attributed to the presence of polymer ions.^27^

The morphology present in the composite, using 5% PVA, confirmed the efficiency of the methodology proposed in this work to obtain a composite with porous morphology.^33–35^ This morphology is useful for the detection of toxic gases by PAni, as well as for the adsorption of these contaminants.

### Atomic force microscopy – AFM

**Fig. 6:**
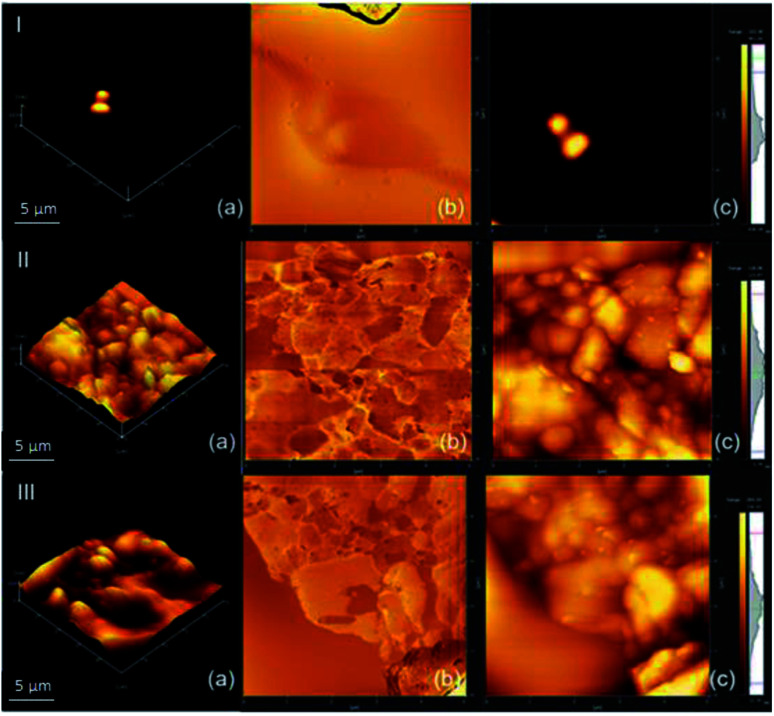
AFM of the composite of (I) NPIO, (II) PAni and (III) PAni/NPIO 20%: (a) 3D morphology, (b) phase analysis and (c) 2D topography.

Fig. 6 shows the composite morphological analysis by AFM. Fig. 6(a) shows a 3D map of the surface morphology over a small surface area (5 × 5 μm^2^) with the presence of valleys, which can be attributed to the formation of pores. This corroborates the results obtained by SEM, justifying the use of the proposed methodology for the application of the system in the sorption of gases.

The AFM technique is scarcely reported in the literature for the materials used in this work. However, studies on the preparation of composites containing PAni showed the presence of two phases, with the insertion of the polymer in the system.^36,37^ According to the literature, PAni presents a densely compacted morphology,^33,38^ which could be observed by the micrographs, and which corroborates the results obtained by SEM. It is possible to observe in Fig. 6(c) the presence of 3 distinct colors, which can be attributed to PAni, on the surface, PVA dispersed in the medium, as well as magnetic nanoparticles, in the darkest parts, since polymers covered them.

### Magnetic properties studies

#### Magnetic force analyses

**Fig. 7:**
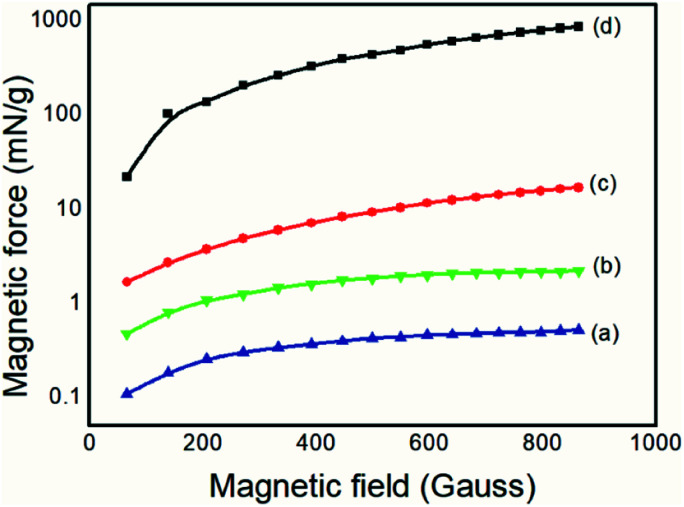
Magnetic forces of (a) PAni, (b) PAni/NPIO 20% composites, (c) NPIO, (d) IO.

**Fig. 8:**
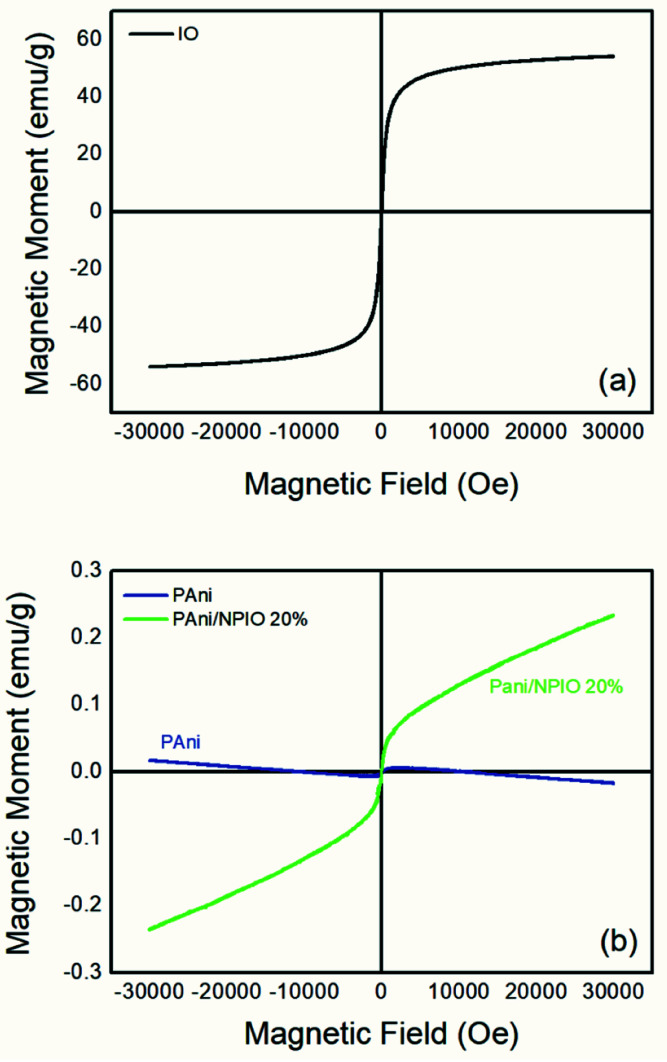
Magnetization curves of (a) IO and (b) pure PAni and the PAni/NPIO 20% composite.

In order to confirm the presence of magnetic nanoparticles, magnetic force and magnetization analyses were performed on each material. Fig. 7 shows the results of the magnetic force as a function of the applied magnetic field. Fig. 8 shows the results of the magnetization analysis.

The magnetite produced, for comparative purposes, presented a magnetic force of 867.71 mN g^−1^, which was above the expected value already reported in the literature.^37^ For the colloidal dispersion of magnetic nanoparticles, a value of 16.97 N g^−1^ was obtained, below that of the magnetic particles. However, both the magnetic nanoparticles and the composite containing 20% of the magnetic nanoparticles had values of magnetic force that were 104 and 10 orders of magnitude higher than the pure PAni, respectively, thus proving the presence of the magnetic material, as expected.

### Magnetization analyses

The magnetization describes how the material with magnetic properties reacts to an applied external magnetic field based on the alignment of their magnetic moment.^39,40^Fig. 8 shows the magnetization curves of magnetization *M*, *versus* the applied magnetic field H for IO particles, pure PAni and the composite at room temperature under magnetic fields of 30 kOe.

In the IO particles, Fig. 8a, magnetization saturation occurred at a relatively low external field of approximately 3300 Gauss, with a value of 55.27 emu g^−1^ according to the literature.^41^ It is possible to observe zero coercivity and zero remanence on the magnetization curve, indicating the superparamagnetic behavior of the magnetite nanoparticles.^42^ For PAni, a non-magnetic moment aligned with the magnetic moment was observed (Fig. 8b), with 0 emu g^−1^ as expected.^39^ This is due to the presence of polyaniline.^42^ For the composite PAni/NPIO 20% the intermediate magnetization curve was observed as expected, indicating the presence of both materials in the composite.

### Gas exposure analysis

#### BET method – multimolecular adsorption analysis – ASAP

**Fig. 9:**
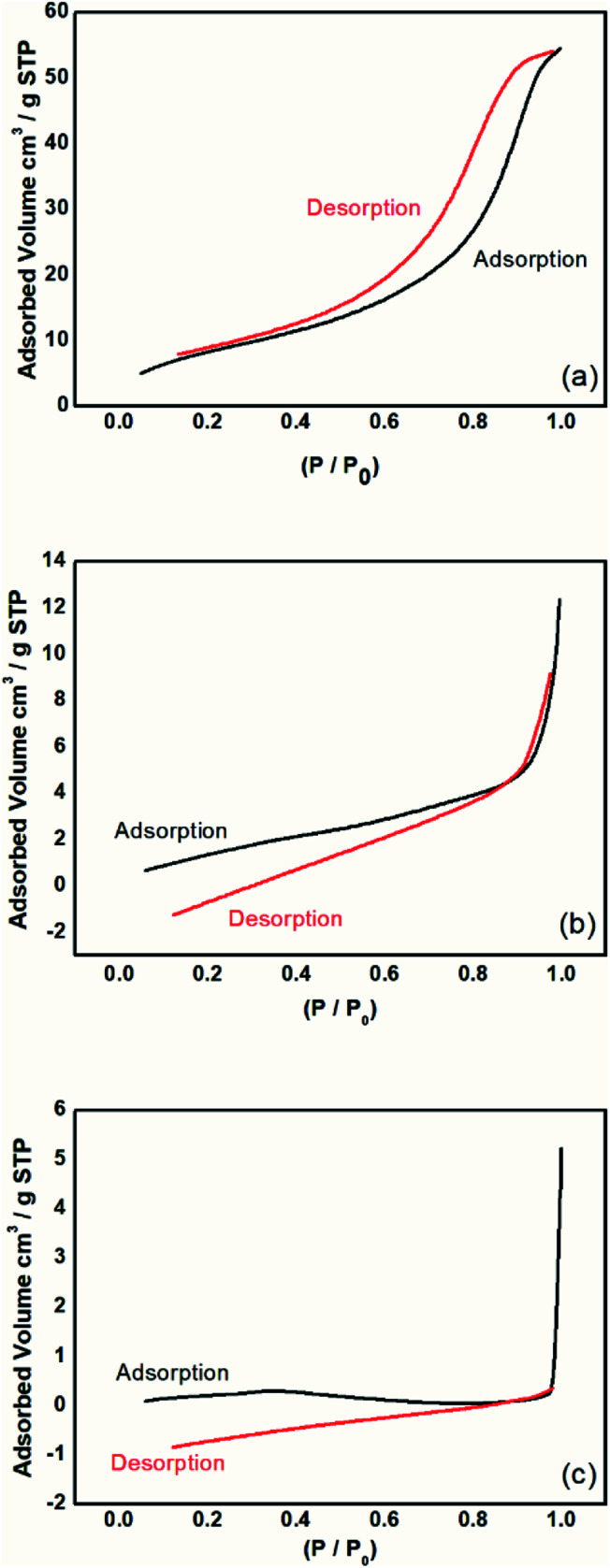
Isotherms of adsorption and desorption analysis by the BET method for the (a) magnetic particles, (b) PAni, and (c) PAni/NPIO 20%.

Analyses of surface volume and pore size were performed on the materials *via* adsorption isotherms using the BET method. Fig. 9 shows the adsorption and desorption behaviors of the samples.

The magnetic particles had a surface area of 31.8 m^2^ g^−1^ with a calculated pore volume of 0.08 cm^3^ g^−1^ and average size of 116.16 Å. PAni and PAni/NPIO 20% presented surface area values equal to 6.48 m^2^ g^−1^ and 5.50 m^2^ g^−1^, with pore volume of 0.01 cm^3^ g^−1^ and 0.003 cm^3^ g^−1^ and diameters of 117.90 Å and 25.75 Å, respectively. Fig. 6 shows the adsorption and desorption tests of the materials in a N_2_ flow.

The observed adsorption isotherms are of type IV, according to IUPAC.^43^ Thus, the adsorbents possess pore sizes of diameters between 2 and 50 nm, which are mesoporous.^44^ This behavior indicates that the relative pressure associated with the amount of gas in the system varies a little, the adsorbed volume increases substantially, and the process is reversible.^39^ This information confirms that the materials present gas adsorption characteristics and are applicable to the objective of this work.

It was also observed that the materials showed adsorption hysteresis, whose desorption does not coincide with the adsorption type H1, which indicates that there is a narrow distribution of relatively uniform pores with cylindrical shape.^43^ The presence of hysteresis also indicates that the materials, besides having pores, have pores with small sizes. The behavior identified by the isotherms proves the presence of pores, indicating the methodology efficiency proposed in this paper and corroborates the information obtained in the morphological analysis of the material.

Although the surface area, as well as the volume of gas adsorbed to the composites, has decreased, it should take into account the complex material system whose application is not limited only to adsorption by magnetic nanoparticles, but also includes the detection of gas by the PAni doping process, a gas fraction that will be retained in the polymer matrix.

### Proposed model for the detection/sorption/filtering process

**Fig. 10:**
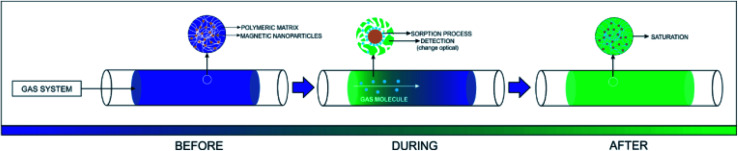
Representation of the device exposure to sulfide gas.

Fig. 10 shows the representation of the operation of the presented system. The inserted gas caused a change in the optical and electrical properties of PAni due to the doping process, as well as the electrical percolation of the polymer.

The PAni doping process is due to the formation of ions and counterions in the quinoid and benzoid units,^6^ forming bipolarons that are converted to polarons^45^ by the effect of the resonance of the electron.^46,47^ The doping also produces a change in the PAni optical properties. More specifically, its conductive form is greenish, while the non-conducting one is blueish.^48,49^ Concomitantly, the iron nanoparticles also participate in the adsorption process since the H_2_S molecules react with the OH groups on the surface of the inorganic material, forming iron III sulfide (Fe_2_S_3_). The presence of moisture in the system also plays an important role. The H_2_O promotes the dissociation of H_2_S into the sulfide ions (HS^−^), which can replace the OH groups on the surface of the inorganic phase.^50^ Finally, iron oxides, especially magnetite, present an expressive density of O^2−^ in their structure. After the consumption of the OH groups, the O^2−^ species interacted with the H_2_S molecules, reducing the Fe III complexes, leading to the formation of SO_2_.^51,52^ Thus, multiple mechanisms can act together in the proposed material, improving, in theory, the efficiency of the capture of toxic vapors.

### Gravimetric analyses

**Fig. 11:**
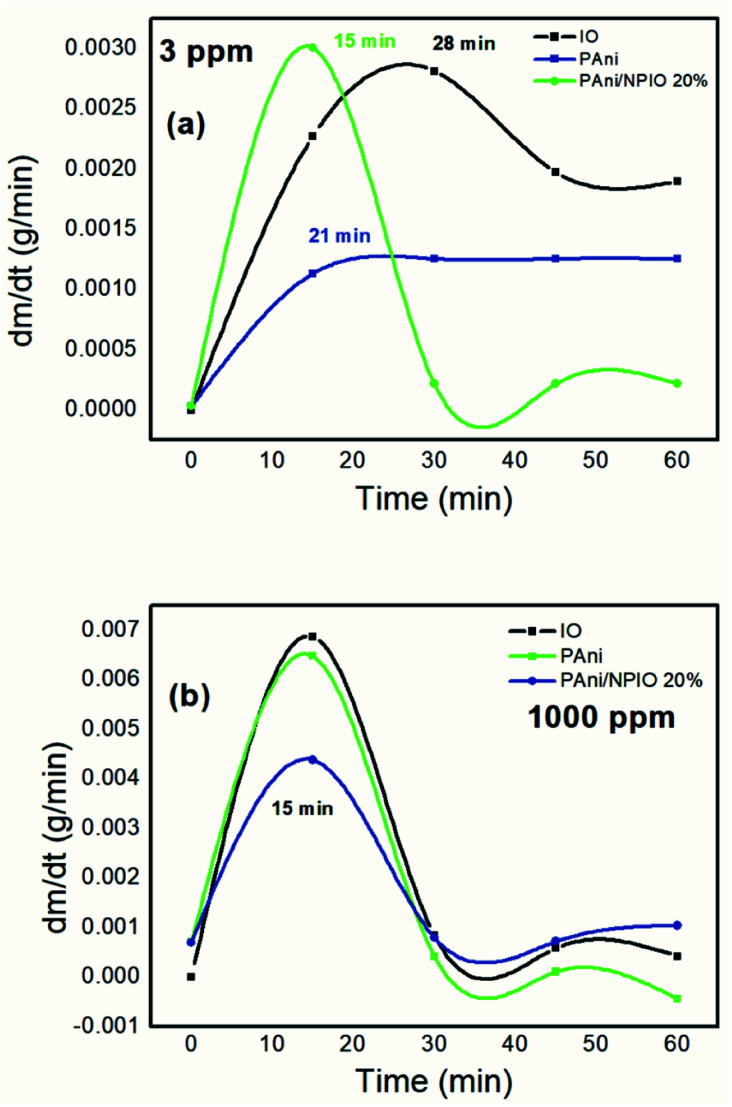
First derivative of the gravimetric curves of the materials exposed to 3 (a) and 1000 ppm (b) of H_2_S.

The gravimetric analyses were performed, aiming to determine the mass variation of the materials over the experiment time. Fig. 11 shows the first derivative of the gravimetric curves during exposure to H_2_S gas at 3 and 1000 ppm for 60 minutes.

The maximum sorption rate indicated that in the first 15 minutes, for the 3 ppm gas concentration, the composite PANI/NPIO 20% showed more significant adsorption in comparison with pure PANI and IO, proving the efficiency of the material. On the other hand, for the tests at 1000 ppm, all the materials presented the same saturation time of around 15 minutes. This behavior is due to the elevated concentration of the gas, which quickly saturated all the systems. However, the established levels for humans to be exposed without causing damage is below 10 ppm.^53,54^

### Thermogravimetric studies

**Fig. 12:**
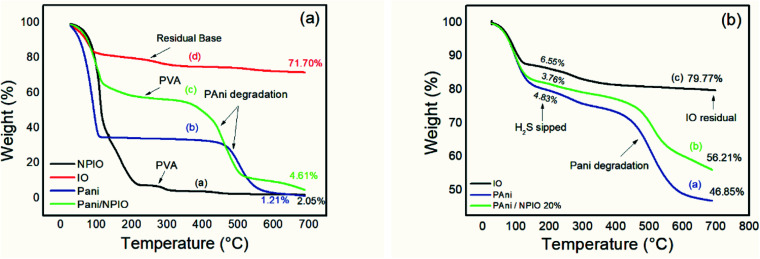
Thermogram of materials (a) before and (b) after exposure to H_2_S with a concentration of 3 ppm.

Fig. 12 shows the TGA of the materials before exposure (a) and after exposure (b) to H_2_S.

Thermogravimetric analyses were performed on the insulating materials and composites before and after exposure to hydrogen sulfide gas. Fig. 12a shows pre-exposure thermal degradations between 100 °C and 200 °C for all materials, most clearly for pure PAni and for the dispersion of magnetic nanoparticles in PVA, which can be attributed to water loss and some components in the pure PAni,^35^ and loss of water for the dispersion of NPIO.

The second thermal decomposition, after 100 °C extending up to 500 °C, was observed in all materials except for pure PAni and the composite. Degradations between 200 °C and 300 °C were attributed to the presence of the dopant;^55^ its absence indicates its withdrawal altogether, or a good part of it, confirming the efficiency of the doping. This process of doping is essential for the application since the acid for the synthesis of the polymer and the acid gas have sulfur in their composition. The third thermal decomposition for the PAni and the composite was attributed to the degradation of the polymer chains.^56^

For the IO particles, NPIO dispersion and the composite, thermal degradations were observed before 200 °C, which can be attributed to the water decomposition and groups corresponding to PVA. A second thermal degradation event, around 200 °C, and 300 °C were also observed and correspond to the dehydration of –OH groups in the polymer chains of PVA present in the dispersion of magnetic nanoparticles. The third thermal decomposition for the NPIO after 300 °C was attributed to the degradation of the PVA on the surface of the magnetic nanoparticles.^57^

**Fig. 13:**
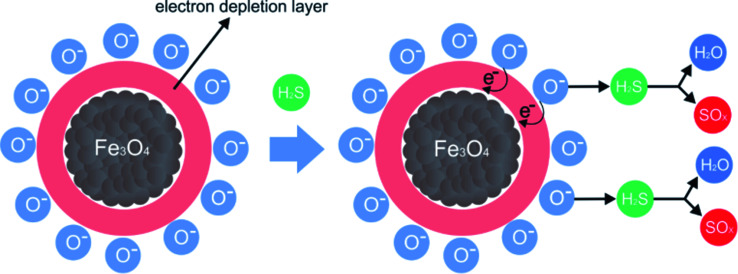
Proposed mechanism for the H_2_S adsorption process using magnetic nanoparticles and forming SO_*x*_ and H_2_S species; adapted from Chen *et al.* 2017.^17^

PAni and the composite presented a thermal decomposition from 200 °C to 300 °C, which was attributed to the doping of the polymer and the composite by the acid gas. In comparison to the analogous non-tested materials, for the materials exposed in this process, when Fe_3_O_4_ is in contact with air, oxygen molecules are absorbed on its surface, forming chemically active species with negative charges and capturing electrons from the conduction layer, which forms an electron-depleting layer (Fig. 13).

When the gas comes into contact with magnetic nanoparticles, negatively charged oxygen molecules form species of sulfur oxide and water.^51^

### Optical properties studies – ultraviolet spectroscopy in the visible region (UV-vis)

**Fig. 14:**
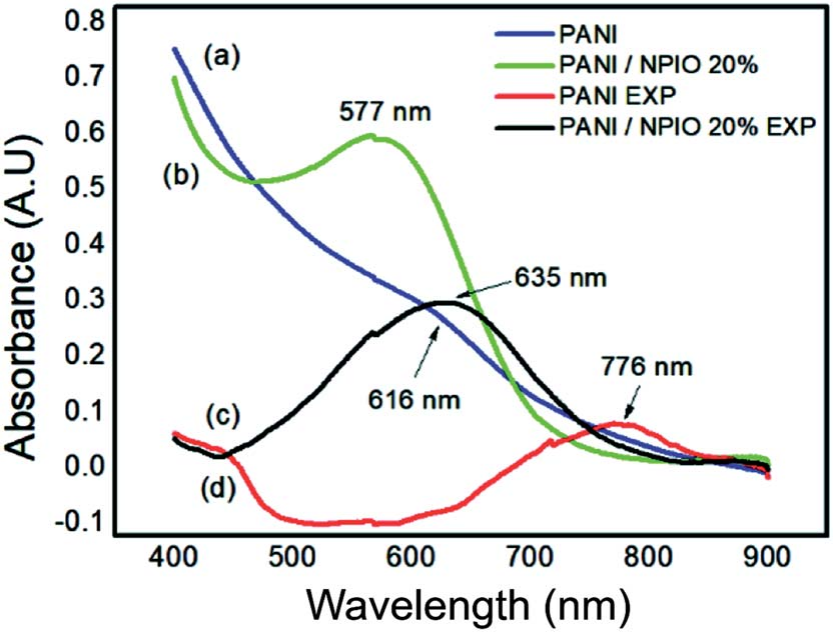
UV-vis spectra of (a) pure PAni and (b) the composite before exposure and (c) pure PAni and (d) composite after exposure to H_2_S.

To identify changes in the optical properties of PAni, UV-vis analyses of the PAni and composite were performed before and after their exposure. Fig. 14 shows the corresponding absorptions of each material.

Absorbances at 577 nm and 616 nm were observed for the composites and PAni. They correspond to the π–π* transitions, of the non-conductive PAni.^57^ The shift to longer wavelengths indicates that PAni was obtained in its doped state.^29^ The observed shifts in the absorptions of PAni and the composite confirmed the changes in the optical properties of the materials, proving that they can be used as sensors for the detection of H_2_S gas.

### Electrical resistivity

Table 2 shows the values of the electrical resistivity of the materials before and after exposure to hydrogen sulfide.

**Table 2:** Electrical resistivities of the magnetic particles of IO, PAni and the PAni/NPIO 20% composite

Sample	Electrical resistivity (Ω cm)	
	Before exposure	After exposure
IO particles	1.0 ± 0.1 × 10^9^	5.5 ± 0.7 × 10^6^
PAni	9.8 ± 0.6 × 10^8^	2.7 ± 0.4 × 10^6^
NPIO/PAni	9.8 ± 0.5 × 10^8^	2.8 ± 0.4 × 10^6^

For pure PAni and the PAni/NPIO 20% composite, in its insulating form, emeraldine base, a high electrical resistivity value was observed, equal to (9.8 ± 0.6) × 10^8^ Ω cm and (9.8 ± 0.5) x 10^8^ Ω cm, respectively. This high electrical resistivity value for PAni in its nonconductive form is attributed to the removal of charge carriers, called ions and counter ions, which reduce the gap between the conduction band and the valence band, which characterize the polymer as a conductor; as these load carriers were removed, the electrical resistivity value increased.^46^ The iron oxide particles presented electrical resistivity equal to (1.0 ± 0.1) x 10^9^ Ω cm. The value of the electrical resistivity can be attributed to the existence of lattice disorder or vacancies that affect the conduction mechanism.

After exposure to hydrogen sulfide, an increase in the electrical resistivity value of the magnetic particles was observed. Semiconductor metal oxides in gases tend to undergo changes in their electrical resistivities^37,50^ due to the reduction of these gases by the oxidative interactions with chemically adsorbed, negatively charged oxygen. When the interactions occur between the oxygen of the iron oxide and the acid gas, the Fe^2+^ ions are oxidized to Fe^3+^, forming a layer of electron depletion and promoting the appearance of holes in the structure of the solid, which prevents the transport of electrons due to the absence of charge carriers, thus the electrical resistivity of the material increases.

After exposure to the hydrogen sulfide gas, a decrease in the electrical resistivity value of pure PAni was observed from (9.8 ± 0.6) × 10^8^ Ω cm to (2.7 ± 0.4) × 10^6^ Ω cm. For the composites of PAni/NPIO, a decrease in the resistivity value was observed, (9.8 ± 0.5) × 10^8^ to (2.8 ± 0.4) × 10^6^. Such a drastic change in the electrical properties of the polymer is attributed to the doping process in the PAni structure, caused by the acid gas.

### Energy dispersive X-ray

**Fig. 15:**
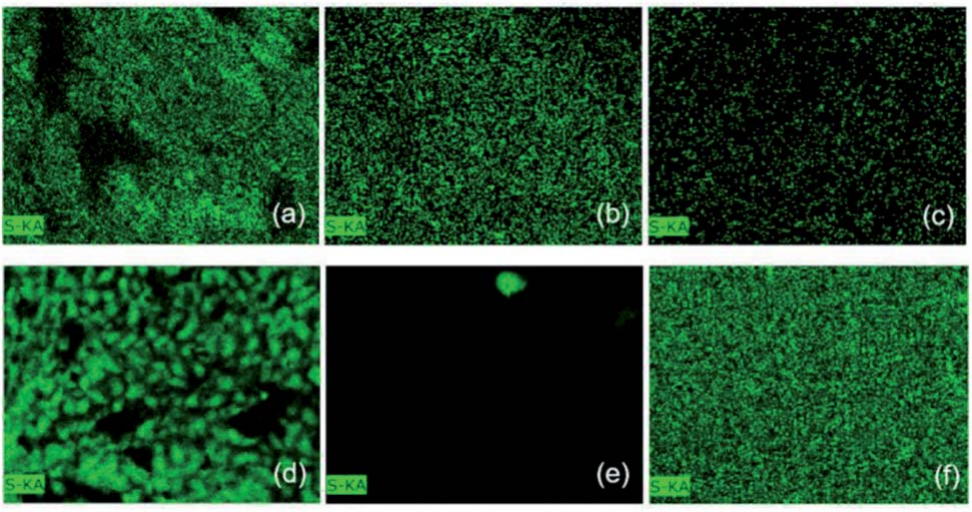
Elementary mapping of the S in (a) magnetic particles, (b) pure PAni and (c) the PAni/NPIO 20% composite before exposure, and (d) magnetic particles, (e) pure PAni and (f) PAni/NPIO 20% composite after exposure to H_2_S gas with a concentration of 3 ppm.

Qualitative analyses to confirm the presence of sulfur after exposure to the H_2_S gas were carried out in the isolated materials and the composite through elemental analysis and mapping by EDX. Fig. 15 shows the mapping of the sulfur element in the materials before and after exposure to the gas. Table 3 shows the normalized elemental composition of the materials, as well as the percentage gain of sulfur before and after exposure.

Elemental composition of materials before and after exposure to H_2_S gas

**Table:** 

Mass% (normalized) before exposure			
	OF	PAni	PAni/NPIO
Nitrogen	0.00	90.54	73.30
Iron	95.35	0.00	13.22
Sulfur	4.65	9.46	13.48

**Table:** 

Mass% (normalized) after exposure			
	OF	PAni	PAni/NPIO
Nitrogen	10.57	69.74	37.79
Iron	38.25	0.05	5.40
Sulfur	51.18	30.21	56.87
Gain% sulfur	46.53	20.75	43.39

EDX proved a significant increase in the amount of sulfur in all the materials. This result is in complete agreement with others presented in this work. The magnetic nanoparticles, PAni, and the composite presented 4.65, 9.46, and 13.48% of S before the test. The presence of sulfur in the materials is due to the reactants used in their preparation. After the sorption test, the same materials presented 51.18, 30.21%, and 56.87% of S in their samples, respectively. Thus, these results proved that H_2_S promoted changes in the materials, which are explained by the doping process of the pure PAni, gas adsorption by the magnetic particles, and a double mechanism based on the doping and adsorption in the composite.

Compared with the conventional sensor available in the industry, the system presented in this work has good sorption and detection in low concentrations and shorter times, and the detection is an essential parameter for sensors. It also has filter characteristics with gas retention in the system, as well as reversibility, making the material reusable.

## Conclusions

A composite with characteristics for the detection and adsorption of H_2_S gas was obtained by joining the electrical/optical properties of PAni and magnetic nanoparticles. The nanoparticles were characterized by light scattering and TEM. The porosity in the polymer matrix was determined by adsorption and desorption isotherms and were analyzed by SEM and AFM. Through the testing of the composite exposure to H_2_S gas, in extreme concentrations, it was possible to identify a good response of the material at low gas concentrations and a lower exposure time below 15 minutes. The response of the material to the gas was verified by employing gravimetric analysis, thermogravimetric, optical, and electrical analyses, with the alteration of the intrinsic properties of PAni. The elemental analysis of the material before and after the exposure, indicated a dramatic increase in sulfur in comparison to the non-exposed PAni, which can be attributed to the doping process of the conducting polymer and sorption by the magnetic nanoparticles. At H_2_S concentration of 3 ppm, the maximum sorption on the derivative showed an increase for the composite, confirming its sorption/detection capacity. The analyses before and after exposure to gas proved that both materials had a positive influence on the application proposed herein. This work has shown that the proposed material has the potential for application in the detection and sorption of toxic gases, especially H_2_S, which was the study gas for the development of the sensor.

## Conflicts of interest

There are no conflicts to declare.

## Supplementary Material
